# Disparities in the accuracy of reporting opioid overdoses to 9-1-1 by race and sex of overdose victim, Marion County, Indiana, 2011–2020

**DOI:** 10.1186/s40352-024-00279-4

**Published:** 2024-05-31

**Authors:** Danielle N. Atkins, Brandon del Pozo, MH Clark, Barbara Andraka-Christou, Daniel O’Donnell, Bradley Ray

**Affiliations:** 1 Askew School of Public Administration and Policy, Florida State University, Tallahassee, USA; 2 The Warren Alpert Medical School of Brown University, Providence, USA; 3 Department of Learning Sciences and Educational Research, University of Central Florida, Orlando, USA; 4 School of Global Health Management and Informatics, University of Central Florida, Orlando, USA; 5 Indiana University, Bloomington, USA; 6 RTI International, Research Triangle Park, USA

**Keywords:** Overdose, Disparities, Emergency medical services, Naloxone

## Abstract

**Objectives:**

To assess the prevalence of emergency medical incidents wherein naloxone was administered but overdose was not described as the chief complaint during the 9-1-1 call, including differences by overdose victim race/ethnicity and sex.

**Methods:**

We computed the percentage of 9-1-1 calls in Marion County, Indiana, from 2011 to 2020, wherein naloxone was administered but the caller did not describe overdose as the chief complaint. We estimated a logistic regression to examine the associations between reporting of overdose as the chief complaint and race and sex of the overdose victim.

**Results:**

Almost one-fifth of 9-1-1 calls preceding naloxone administration did not describe overdose as the chief complaint. 9-1-1 callers were more likely to describe a non-overdose as the chief complaint when the overdose victim was Black or female.

**Conclusion:**

9-1-1 callers are less likely to use terminology describing overdose when the overdose victim is female or Black, than when the victim is male or White. Inaccurate terminology when calling 9-1-1 could delay naloxone administration, thereby increasing risk of overdose death and hypoxic brain injury. Some 9-1-1 callers may be avoiding overdose terminology to prevent a police response, or due to lack of knowledge about overdose identification, but further research is needed to determine the mechanisms underlying these findings.

## Introduction

The United States is facing an unprecedented fatal overdose crisis (NCHS, 2023), with opioids involved in 74.8% of total annual overdose deaths (CDC, 2022). Naloxone, a medication that is easy to administer and now available over the counter without a prescription, can effectively reverse opioid overdoses. Time is of the essence during an opioid overdose. Delays in naloxone administration not only decrease the odds of an overdose victim’s survival but can also increase the risk of hypoxic brain injury, organ damage, and resulting cognitive and physical disabilities if the overdose victim survives. ^3^

Despite these concerns, little is empirically known about how callers report overdoses to 9-1-1 in practice. Additionally, given increasing racial and ethnic disparities in overdose deaths (Kariisa et al., 2022), understanding differences in overdose reporting to 9-1-1 by victim demographics is of critical importance. Previous research has found that 9.3% of emergency medical response services for overdoses in Indianapolis, Indiana were associated with the subsequent arrest of the overdose victim (Ray et al., 2022), giving credence to the idea that people may seek to avert a police response when they call for help in settings with a notable prevalence of arrest. We are unaware, however, of studies examining the terminology used by 9-1-1 callers at overdose scenes, and if vague reports are more prevalent among overdose victims of particular demographics. To begin to fill this gap, wobtained 10 years of EMS records in Marion County, Indiana where medical responders administered naloxone prior to the victim’s arrival at a hospital, indicating a diagnosis of possible opioid overdose was made at the scene. We then analyzed these records to ascertain how the incident was initially reported during the call to 9-1-1, whether the principal complaint was of an overdose event, and if there were significant differences in the nature of these initial reports based on the demographic characteristics of the victim.

## Methods

This study analyzed prehospital care report data for 11,570 9-1-1 calls resulting in prehospital administration of naloxone by Marion County, Indiana first responders from 2011 to 2020. Marion County is 99.1% urban (Purdue, 2020), and wholly contains Indianapolis, which had a population of 887,642 at the time of the 2020 census (Census, 2023). Utilized in prior published research (Ray et al., 2022, 2023), the data included the chief and secondary complaint provided, overdose victim’s sex, and overdose victim’s race/ethnicity. Overall and by demographics, we computed the percentage of 9-1-1 calls where a witness did not report a chief or secondary complaint of overdose, but first responders ultimately administered naloxone. We estimated a logistic regression to examine the associations between overdose victim’s sex and race and the likelihood of describing overdose as the complaint. All analyses were conducted using Stata v. 17 (StataCorp, 2021). Our study was approved by the Lifespan IRB.

## Results

Of the 11,570 patients administered prehospital naloxone, 58.0% were male, 42.0% were female, 21.2% were Black, 77.4% were White, 0.4% were Hispanic, and 1.0% were of other races (Table 1). Almost one-fifth (18.4%) of the naloxone administration incidents began with a complaint to 9-1-1 other than overdose. 9-1-1 callers were significantly less likely to describe a non-overdose event when the victim was male as compared to female (OR = 0.90; CI [0.82, 0.99]; *p* < 0.05). 9-1-1 callers were significantly more likely to describe a non-overdose as the complaint when the overdose victim was Black as compared to White (OR = 2.58; CI [2.33, 2.87]; *p* < 0.001) (Table 2). There were no significant interactions between race/ethnicity and sex.

**Table 1:** Descriptive Statistics of 11,570 patients who were administered prehospital naloxone by first responders in Marion County, Indiana, 2011–2020

	(1) Full Sample Mean
Non-overdose call	18.4%
Male	58.0%
Female	42.0%
Black	21.2%
White	77.4%
Hispanic	0.4%
Other	1.0%
Observations	11,570

**Table 2:** Association between patient characteristics and the likelihood a call began with a complaint other than overdose to 9-1-1

	(1) Non-overdose Call
Male	0.90*
	[0.82,0.99]
Black	2.58***
	[2.33,2.87]
Hispanic	1.24
	[0.60,2.56]
Other	1.55
	[0.98,2.44]
Observations	11,570

*Note* Exponentiated coefficients. Female is the reference category for sex. White is the reference category for race/ethnicity

* *p* < 0.05, ** *p* < 0.01, *** *p* < 0.001

## Discussion

In this cross-sectional study of prehospital naloxone administration, 9-1-1 callers were 2.6 times more likely to initially report the incident as a non-overdose if the overdose victim was Black than if the overdose victim was White. We also found that 9-1-1 callers were significantly less likely to report the incident as a non-overdose if the overdose victim was female. To our knowledge this is the first study to assess the likelihood of 9-1-1 callers using overdose terminology at presumed scenes of overdoses (as indicated by subsequent naloxone administration). Given the magnitude of the opioid overdose crisis and the unambiguous benefits of a timely and accurate call to 9-1-1 when a person is overdosing, research is urgently needed to understand what drives vague and inaccurate reports and explains the demographic disparities that exist in their occurrence.

Several reasons for the disparities in using overdose terminology in a 9-1-1 call may exist. First, qualitative research documents the fear of a police response on the part of overdose witnesses, and specifically fear of police response to an overdose among the Black community in Indianapolis (Wagner et al., 2019; Seo et al., 2023). Such fears may be heightened among people calling 9-1-1 on behalf of racial and ethnic minorities, given the history of systematic police bias in the U.S. We hypothesized that some people may avoid mentioning “overdose” when calling 9-1-1, instead opting for vaguer phrases, such as “difficulty breathing” or “unconsciousness,” in the hope that only medical personnel will arrive at the scene. Some harm reduction trainings even encourage people calling 9-1-1 to avoid overdose terminology for these reasons (Healthline, 2023). Other research at the national level has demonstrated that the overdose death rate among Black men ages 65 and older is almost seven times that of non-Hispanic, White men of the same age (Kariisa et al., 2022). Perhaps callers are less likely to report overdose for older victims if they think the signs of overdose are symptoms of an age-related condition. Alternatively, research has shown that Black individuals are less likely to receive harm reduction interventions than White individuals, which may lead to disparities in reporting overdoses (Dayton et al., 2020; Khan et al., 2023).

We find it more difficult to hypothesize about the reasons for a significant difference in reporting by sex given the paucity of research in this regard. Some hypotheses seem promising, however. Among them are differences in the means of consumption of opioids: if females who use drugs are more likely to ingest pills or smoke, rather than inject substances, the evidence of overdose provided by a needle would be missing in their case (Powis et al., 1996). Another possibility is a hesitancy to alert authorities because it may endanger a woman’s custody of her dependent children. There is evidence that such losses are highly traumatic (Janzen & Melrose, 2017), and can increase subsequent risk of overdose (Darlington et al., 2023), but whether fear of an overdose victim losing custody of her children leads to deliberately vague 9-1-1 calls has yet to be systematically investigated.

Further research is needed to understand the mechanism(s) underlying our findings, especially since the policy response depends on the mechanism(s) at play. If fear of police response is driving our findings, then police responses to overdose must be framed as emergency lifesaving efforts, not opportunities to enforce criminal laws (Wood et al., 2021). Importantly, recent studies demonstrate that police equipped with naloxone can arrive at overdose scenes in advance of medical personnel for a substantial number of calls (Pourtaher et al., 2022; White et al., 2022) filling an acute need in rural areas (Wood et al., 2021). However, by excluding police, who may be able to arrive at overdose scenes faster than medical personnel, inaccurate terminology could decrease the likelihood of timely receipt of lifesaving treatment, illustrating a fundamental tension between a caller’s desire to save a life and prevention of arrest/incarceration. Clearly-worded laws and policies can promote the timely and accurate reporting of overdoses to 9-1-1. Good Samaritan laws offer immunity from arrest when police respond to overdoses, but their protections vary widely by state. Except for serious, violent offenses, these laws should give unambiguous criminal immunity to both the caller and the victim, as well as bystanders at an overdose scene. Police departments can prioritize their lifesaving role in advance of changes to the law by discouraging arrests and prohibiting checking people for arrest warrants at overdose scenes through internal policies, training, and good faith action. If callers are less likely to report older victims experiencing overdose symptoms, who are more likely to be Black, as having an overdose to 9-1-1, perhaps more education and training is needed to identify overdose in this population. If disparities in harm reduction training are contributing to the disparities in 9-1-1 calls reported as overdoses, then efforts to address these differences in training should be undertaken. Finally, if female victims are less likely to be reported as overdose victims due to concerns over child welfare response, municipalities need to carefully weigh the costs and benefits of their prevailing approaches for both the mother and the child. Given that overdose death rates are skyrocketing in Black communities (Kuehn, 2022), misreporting an overdose, for whatever reason, when a Black person is suffering a medical emergency only exacerbates the significant health disparities endured by this marginalized population. Future researcher should examine perceptions of people who use drugs or of those who have recently overdosed to further explore the relationship between identity and 9-1-1 call terminology.

This study has several important limitations. Overdose victims do not call 9-1-1 themselves, and we do not know the demographics of the callers who reported complaints or why some did not report overdoses, although we suspect that in many instances the caller is trying to avoid a police response, along with the other factors highlighted above, such as lack of knowledge or making presumptions based on the age of the victim. It is possible that the caller does not realize the victim is experiencing an overdose. However, it is unlikely that this type of circumstance would prevail among Black and female overdose victims disproportionately enough to explain the significant differences in our reported odds ratios. Also, while we are assuming that an overdose did indeed occur if naloxone was administered, it is also possible that overdose was misdiagnosed by first responders in some cases, and if there are disparities in naloxone administration by race, this would bias our results.

Lastly, this study draws its conclusions by an analysis of one urban county in the US Midwest. We do not know how readily our results would generalize to other settings. For example, in the introduction we discuss the lifesaving advantages of a police response to overdose in rural settings, where the response time of medical personnel can be much longer due to inherently greater distances and fewer emergency resources. However, only further research can assess if the results we observed in Marion County, Indiana prevail in these rural settings. While there is no cause to think they would not, we advise caution in assuming they do.

## Conclusion

Accurate reporting of overdose emergencies to 9-1-1 is a public health priority, particularly in Black communities where overdose death rates are rising. Yet, in the setting studied here, many callers to 9-1-1 either deliberately or inadvertently provided vague or incorrect assessments of a condition ultimately treated by first responders as an opioid overdose, and the risk of such misreporting was significantly higher for Black overdose victims.This research provides critical preliminary evidence identifying a life-threatening issue, specifically among the Black community and female overdose victims. The results call for further research to identify the mechanisms at play to inform an effective policy response, since they highlight ways in which our response to substance use disorder, especially in terms of its punitive focus, continues to cost lives and exact disparate consequences on the nation’s minoritized and vulnerable populations.

## Data Availability

The datasets generated and/or analyzed during the current study are not publicly available because individual privacy could be compromised.
